# Reversal of Azithromycin Resistance in *Staphylococcus saprophyticus* by Baicalin

**DOI:** 10.3389/fvets.2022.827674

**Published:** 2022-02-18

**Authors:** Jinli Wang, Jinwu Meng, Jinyue Zhu, Tianxin Qiu, Wenjia Wang, Jinxue Ding, Zhenguang Liu, Kun Li, Deyun Wang, Jiaguo Liu, Yi Wu

**Affiliations:** MOE Joint International Research Laboratory of Animal Health and Food Safety and Traditional Chinese Veterinary Medicine Research Center, College of Veterinary Medicine, Nanjing Agricultural University, Nanjing, China

**Keywords:** baicalin, *S. saprophyticus*, azithromycin, resistance, reversal

## Abstract

In recent years, the efficacy of antibiotics has been threatened by the evolution of bacterial resistance. We previously demonstrated that baicalin (Bac) showed synergies with azithromycin (Azm) against Azm-resistant *Staphylococcus saprophyticus* (ARSS). The aim of this study was to explore the roles of Bac in reversing the resistance of ARSS to Azm. The ARSS was sequentially passaged for 20 days with the sub-MIC (minimum inhibitory concentration) of Bac. The strain that recovered sensitivity to Azm was named Azm-sensitive *S. saprophyticus* (ASSS). The sub-MIC of Bac reversed the resistance of ARSS to Azm. The MIC of Azm against the ASSS strain was 0.488 mg/l, and it was stable within 20 passages. The highest rate of resistance reversal reached 3.09% after ARSS was exposed to 31.25 mg/l Bac for 20 days. Furthermore, semiquantitative biofilm and RT-PCR assays reflected that the ability of biofilm formation and the transcript levels of *msrA, mphC*, and virulence-associated genes in the ASSS strain were significantly lower than those of the ARSS strain (*p* < 0.05). Simultaneously, Azm delayed the start time of death, alleviated the injury of the kidney, and decreased the bacterial burden in organs and cytokine levels in mice infected with ASSS. In contrast, Azm did not have a good therapeutic effect on mice infected with ARSS. Therefore, Bac has the potential to be an agent that reversed the resistance of ARSS to Azm.

## Introduction

*Staphylococcus saprophyticus* (*S. saprophyticus*), coagulase-negative coccus, causes urinary tract infections (1), francolin ophthalmia (2), and bovine mastitis (3). In poultry, macrolide antibiotics have often been used to treat infections induced with staphylococcus and streptococcus (4). It is reported that 60% of coagulase-negative staphylococci (CNS) isolated from various samples including central venous catheter tips, urine, and blood were highly resistant to penicillin (90%), ceftriaxone (40%), co-trimoxazole (60%), and azithromycin (Azm) (60%) (5). Many resistance genes of macrolides are parts of either transposon, plasmids, phages, or genomic islands and, as such, can easily transfer across species, strain, and genus boundaries (6). Researchers are increasingly identifying multidrug-resistant *S. saprophyticus* from ready-to-eat food of animal origin (7). The presence of resistance in food could be a severe threat to public health due to the possible spread of antibiotic resistance (7). However, resistance rates continue to rise, and the rate of antibiotic discovery has decreased substantially. In recent years, the combination of antibiotic drugs and non-antibiotics for bacterial infection treatment appears promising (8, 9). However, drug-resistant bacteria remain a severe threat to the efficacy of antibiotics. One strategy to deal with this problem was to recover the sensitivity of old antibiotics to bacteria. The methods of preventing the spread of antibiotic resistance need to be prioritized (10, 11).

Regulation of virulence factor production is essential for bacterial colonization and pathogenesis (12). Virulence inhibitors are important therapeutic means to treat bacterial infections (13). Virulence inhibitors only inhibit the colonization and virulence of bacteria without exerting selective pressure to hinder the emergence of antibiotic resistance (14). In *S. saprophyticus*, urease (ureC), Uro-adherence factor (UafA), autolysis Aas, surface-associated protein of *S. saprophyticus* (ssp), and D-serine deaminase (dsdA) are associated with colonization and pathogenicity (1, 15, 16). The biofilm of *S. saprophyticus* could also cause persistent infection. Therefore, the development of effective virulence inhibitors is a potential method to deal with bacterial resistance.

Baicalin (Bac) is a potential candidate for reversing Azm resistance in Azm-resistant *S. saprophyticus* (ARSS), a flavonoid compound, extracted from *Scutellaria baicalensis* Georgi (17). It has been reported to restore the effectiveness of β-lactam antibiotics against β-lactam-resistant staphylococcus (17). We previously demonstrated that Bac combined with Azm exhibited synergistic activity against Azm-resistant *S. saprophyticus* (ARSS) (2). Although the synergy of Bac with Azm against ARSS has already been reported, its ability to reverse Azm resistance of ARSS and inhibit virulence has not previously been scrutinized.

Based on this information, we hypothesized that Bac had the potential to reverse the resistance of ARSS to Azm. Thus, the serial passage of ARSS exposure to the sub-MIC (minimum inhibitory concentration) of Bac was conducted. Moreover, the treatment efficacy of Azm to mice infected with the strain that recovered sensitivity to Azm by the serial passage was detected.

## Materials and Methods

### Strains and Culture Conditions

Azm-resistant *S. saprophyticus* (Azm MIC of 1,000 mg/l determined by the broth micro-dilution method) was isolated from francolins suffering from ophthalmia in a francolin farm located in Jiangsu province, China (2). Furthermore, strains were cultured in Mueller–Hinton broth (MHB) or nutrient broth (NB, Hopebio, Qingdao, China) at 37°C with shaking at 180 rpm.

### The Rate of Resistance Reversal

For the resistance reversal assay, 10^6^ colony-forming unit (CFU) exponential-phase ARSS was inoculated into 1 ml of MHB containing Bac at sub-MICs: 250, 125, 62.5, 31.25 mg/l. After 24 h, the cultures allowed growth were further diluted at 1:100 with fresh MHB containing Bac. The passaging process was repeated for 20 days consecutively. Every five passages, approximately 1 × 10^3^ CFU ARSS strain exposed to sub-MIC levels of Bac were cultured on mannitol salt agar (MSA) plates at 37°C. After 18 h of incubation, the colonies from the MSA plate were transferred onto a drug-free MSA plate and an MSA plate containing 250 mg/l Azm. Then the plates were cultured for 24 h at 37°C. The numbers of colonies surviving in the plate containing Azm and drug-free plate were respectively recorded as C_1_ and C_2_. Finally, the rate of resistance reversal was calculated based on the formula: resistance reversal rate (%) = (1 – C_1_/C_2_) × 100%. The strains that were recovered sensitivity to Azm were named Azm-sensitive *S. saprophyticus* (ASSS).

### MIC Determination

The ASSS and ARSS strains in the logarithmic growth phase were diluted to a final concentration of 1 × 10^6^ CFU/ml in MHB. Azm and Bac were diluted using a two-fold serial dilution to obtain the target concentration with MHB in a 96-well plate. A total of 50 μl of bacteria suspension was mixed with 50 μl of the compound and incubated aerobically for 18–24 h at 37°C. The lowest concentration which inhibited the visible growth of bacteria was defined as the MIC value. All of the assays were performed in triplicate independently with two samples.

### The Stability of Resistance Reversal

The ASSS and ARSS strains were passaged in MHB without Bac. Briefly, exponential-phase bacteria (10^6^ CFU/ml) were grown in MHB and cultured for 24 h. Then, the cultures were diluted at 1:100 into fresh MHB. The serial passages were conducted daily until the 20th passage. At least three independent biological replicates of each experiment were carried out. The MIC was determined as described as above every five passages.

### Determination of Growth Curve

For the growth kinetic assay of ARSS and ASSS, overnight cultures were prepared and cultured in NB. The cell densities at 600 nm were measured at 0, 2, 4, 6, 8, 10, 12, and 24 h. For the growth curve under Bac, overnight ARSS cultures were prepared and cultured in MHB containing sub-MIC of Bac. The cell densities at 600 nm were measured at 0, 2, 4, 6, 8, 10, 12, 14, and 24 h.

### Gene Transcription Levels Measured by RT-PCR

To measure the mRNA transcript levels of *msrA, mphC, dsdA, ureC, Aas, UafA*, and *ssp* genes, RT-PCR was conducted as previously described (18). The Bacteria RNA Extraction Kit (Angle Gene, Nanjing, China) was used to extract the total RNA from bacterial cells in the mid-log phase. The values of *A*_260_/*A*_280_ were 1.8–2.1. The RNA was used to reverse into cDNA using a HiScript II 1st Strand cDNA Synthesis Kit (Vazyme, Nanjing, China). Thereafter, the reverse transcription was performed at 50°C for 15 min and 85°C for 5 s. RT-PCR reactions were carried out in a StepOne PCR instrument (Applied Biosystems, Foster City, CA, USA) using ChamQ™ SYBR® qPCR Master Mix (Vazyme, Nanjing, China) as recommended by the manufacturer's instructions. The protocol of the RT-PCR reaction was as follows: first holding stage at 95°C for 3 min, followed by a cycling stage at 95°C for 10 s and 60°C for 60 s (40 cycles total), and a final melting curve stage at 95°C for 15 s, then 60°C for 60 s, and 95°C for 15 s. The housekeeping gene *16S rRNA* was chosen as the internal control gene. The method of 2^−ΔΔCT^ was used to analyze the data which were presented relative to the ARSS group. Primer sequences used are shown in Table 1.

**Table 1:** Oligonucleotide primers used in this study.

**Target gene**	**Primer**	**Sequence (5^′^-3^′^)**	**Source**
*16S rRNA*	*16S rRNA*-F	AGTTGTTCTCAGTTCGGATT	This study
	*16S rRNA*-R	ATACGGCTACCTTGTTACG	
*msrA*	*msrA*-F	GCTCTACTGAATGATTCTGATG	This study
	*msrA*-R	TGGCATACTATCGTCAACTT	
*mphC*	*mphC*-F	GAGACTACCAAGAAGACCTGACG	This study
	*mphC*-R	CATACGCCGATTCTCCTGAT	
*dsdA*	*dsdA*-F	GTGGAAGTCATAGAACATCAG	This study
	*dsdA*-R	GCGTCATCATACCTAATAGC	
*ureC*	*ureC*-F	ACACATATCGGTGGCGGTACAG	This study
	*ureC*-R	GGTTTACAGCTTGCCCTTTACCAG	
*Aas*	*Aas*-F	GCCGACTACGCAGCAACTAAC	This study
	*Aas*-R	CCATGAGGGTCAGAGTGGTCAG	
*uafA*	*uafA*-F	TTCGGTGGTTATGTATGGTT	This study
	*uafA*-R	CAGTGTTGTTCGCTTGTG	
*ssp*	*ssp*-F	ACTTCGGTCTATCTCAATGG	This study
	*ssp*-R	ACATCTGTTGCTTCGGTATA	

### Biofilm Assay

Biofilm formation assays were performed following a previously published protocol with slight modifications (19). Briefly, a 96-well plate was inoculated with 1 × 10^5^ CFU/ml ARSS or ASSS suspension (200 μl/well) and incubated at 37°C without shaking. After 24 h, the supernatants were removed, and wells were washed with PBS twice. Afterward, the attached bacteria were fixed by 2.5% glutaraldehyde for 1.5 h and then air-dried at room temperature. The wells were stained by adding 200 μl 1% (wt/vol) crystal violet for 20 min and then rinsed thoroughly with PBS until the negative control wells (without bacteria) became colorless. Finally, 200 μl of 33% glacial acetic acid was added to the wells and the absorbance values were detected at 570 nm using a Thermo™ Multiskan™ FC enzyme-labeled instrument. The data were calculated by subtracting the values in the negative control from all the experimental groups.

### Mouse Model of ASSS or ARSS Infection

For the experiment, 100 female ICR mice (18–20 g) were randomized into five groups: ASSS group, ASSS-Azm group, ARSS group, ARSS-Azm group, and blank control (BC) group. For mice in the ASSS and ASSS-Azm groups, they were intraperitoneally injected with 3.6 × 10^9^ CFU of the ASSS strain in sterile normal saline. The mice in the ARSS and ARSS-Azm group were challenged with 2 × 10^9^ CFU of the ARSS strain in sterile normal saline. In the BC group, mice were incubated with equal-volume normal saline. Two hours later, mice in the ASSS-Azm and ARSS-Azm groups were orally administrated with Azm at a dosage of 75 mg/kg, once a day for 3 days. To ensure consistency across tests, mice in the other groups were treated with equal-volume normal saline. At 10 h postinfection, five mice were anesthetized and blood was collected *via* retro-orbital bleeding. Then, clotted blood was centrifuged at 3,000 rpm for 15 min and serum was collected. The levels of IL-6, IL-8, and TNF-α in serum were measured with ELISA kits (Angle Gene, Nanjing, China). The liver, spleen, kidney, and bladder were removed after thoracotomy, weighed, and homogenized in sterile saline solution. The serially diluted samples were plated onto MSA plates for CFU enumeration. For hematoxylin and eosin (H&E) staining and examination, the kidney was collected and fixed in 4% paraformaldehyde. The mice were monitored for a survival rate up to 58 h.

### Statistical Analysis

Mean ± standard deviation (SD) was used to express the data. Duncan's multiple-range tests and independent-sample tests were performed to analyze the data among groups using SPSS software (IBM SPSS Statistics 20.0). The statistical significance level was *P*-value ≤ 0.05.

## Results

### The Growth Curve of ARSS Under sub-MIC of Bac

**Figure 1:**
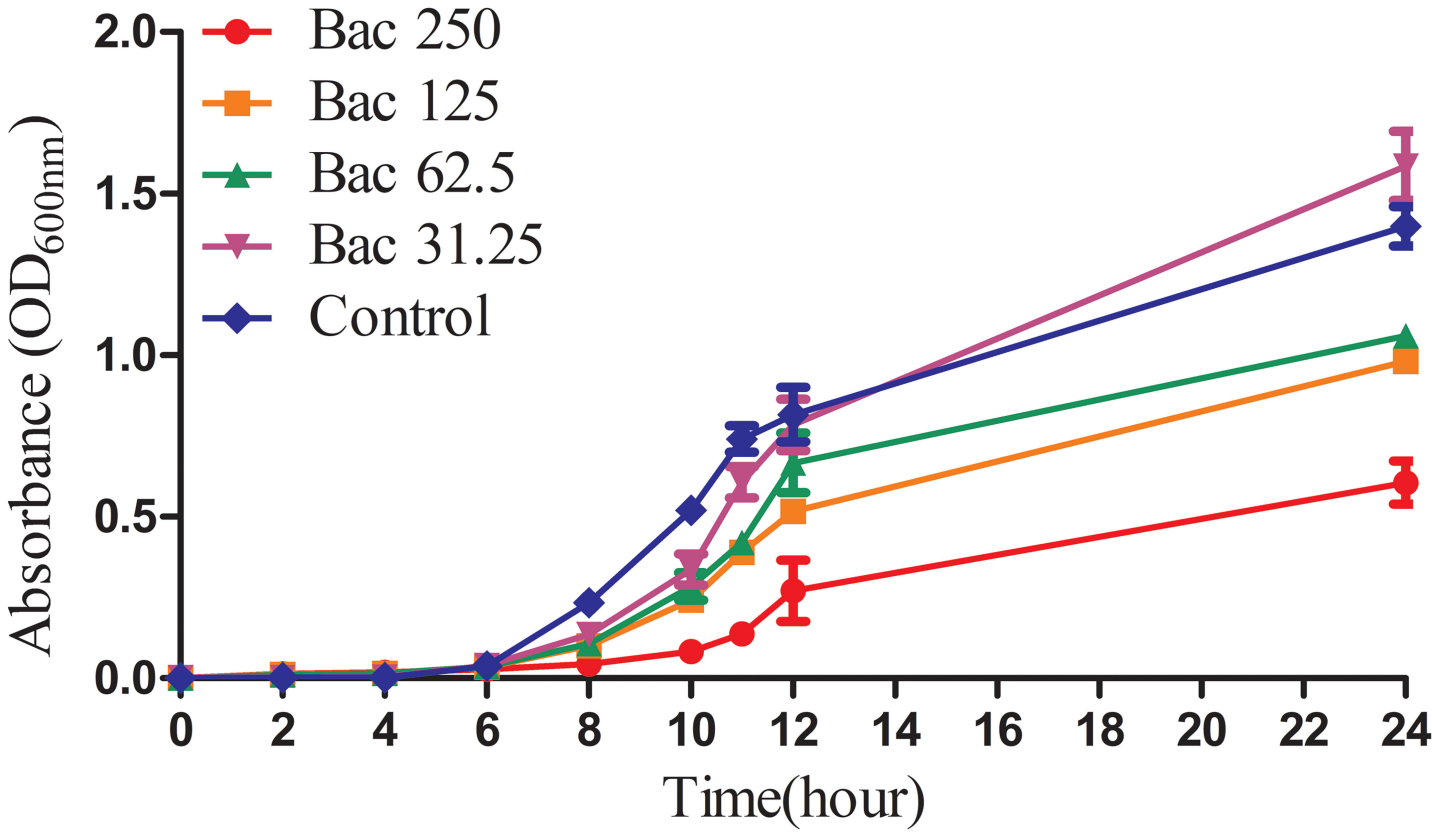
The growth curves of ARSS in MHB under the sub-inhibitory concentration of Bac. OD_600nm_ values were the means ± SD from three independent experiments.

Previously, we indicated that the MIC of Bac against ARSS was 500 mg/l (2). In this experiment, the growth curves of ARSS under sub-MIC of Bac were measured. As shown in Figure 1, 250 mg/l Bac inhibited the growth of ARSS in MHB. The start time of the exponential phase of the 250-mg/l Bac group was 2 h later than the other groups. In the 250-mg/l Bac group, the OD_600nm_ values were three-fold lower than those of the control group at 12 h. 125 and 62.5 mg/l Bac moderately inhibited the growth of ARSS. However, we can detect a marked multiplication of strain in the 31.25-mg/l Bac and untreated groups.

### The Effect of Bac Reversed the Resistance of ARSS to Azm

**Figure 2:**
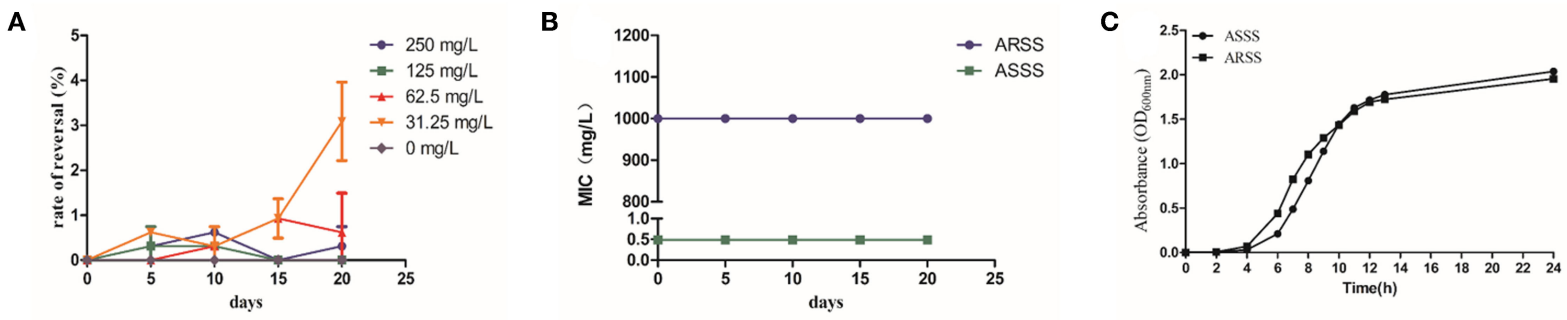
Bac reversed the resistance of ARSS to Azm. **(A)** Rate of resistance reversal development of ARSS to Azm during serial passaging in the presence of sub-inhibitory concentrations of Bac. **(B)** The MICs of Azm against ARSS and ASSS during passaging under non-drug MHB. **(C)** The growth curve of ARSS and ASSS. OD_600nm_ values are the means ± SD. All tests were done with three biological replicates.

As shown in Figure 2A, the rates of resistance reversal gradually increased in the presence of sub-MIC of Bac over time. Importantly, in the 20th passage, the rate of resistance reversal was the highest, reaching 3.09% in the 31.25-mg/l Bac group. The strain that recovered sensitivity to Azm was named ASSS. Then, the MIC and stability of resistance reversal were determined. The MIC of Azm against ASSS decreased 2,049-fold to 0.488 mg/l compared with the ARSS strain. The MICs of Bac against ARSS and ASSS were 500 mg/l (Table 2), and the MICs of Azm against ASSS and ARSS were stable within 20 passages in non-drug MHB (Figure 2B). Simultaneously, the growth curves of ARSS and ASSS were similar in NB (Figure 2C).

**Table 2:** Minimal inhibitory concentrations (mg/L) of Azm and Bac.

**Strain**	**MIC (mg/L)**	
	**Azm**	**Bac**
ARSS	1,000	500
ASSS	0.488	500

*Azm, azithromycin; Bac, baicalin*.

### The Changes of the Transcript Levels of Resistance-Associated Genes and Biofilm Formation Ability

**Figure 3:**
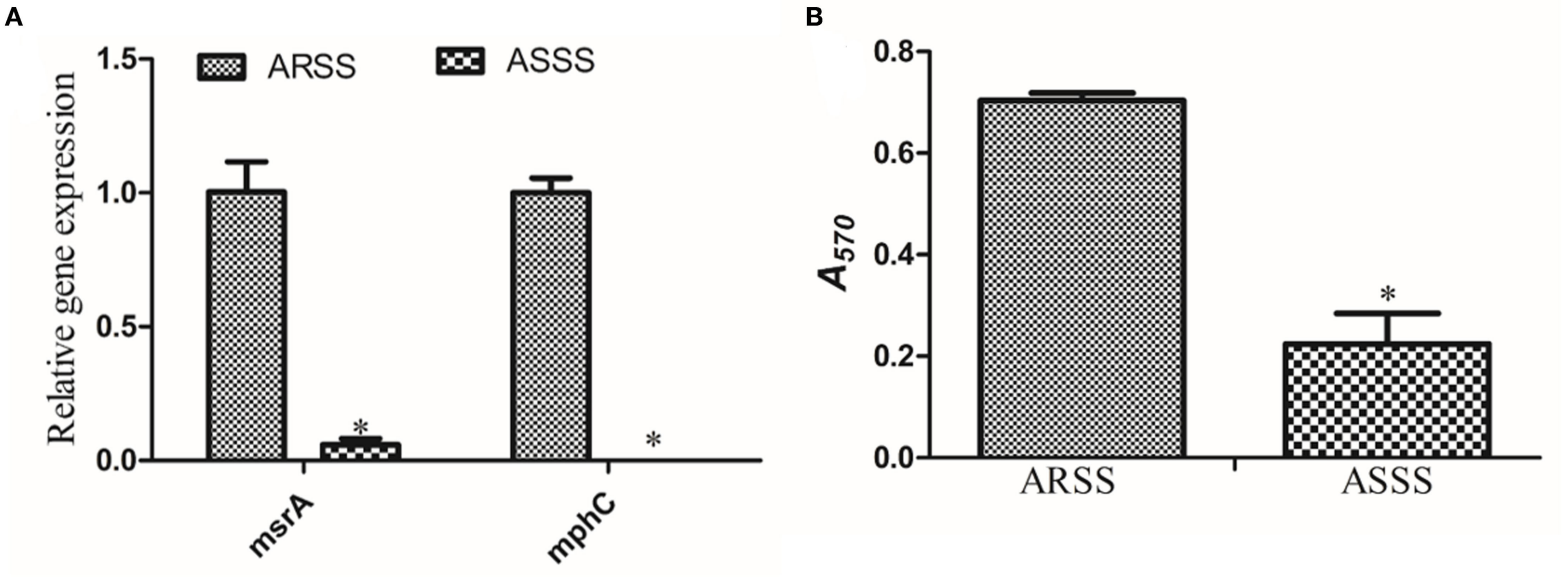
The changes of the transcript levels of resistance-associated genes and biofilm formation ability. **(A)** The transcript levels of *msrA* and *mphC* genes were detected by the RT-PCR method. **(B)** The biomass of ARSS and ASSS was assessed by the crystal violet assay. *p < 0.05.

To measure the mRNA transcript levels of resistance-associated genes in ARSS and ASSS, RT-PCR was performed. As shown in Figure 3A, the mRNA transcript levels of *msrA* and *mphC* genes in the ASSS group were prominently lower than those in the ARSS group (*P* < 0.05). Biofilm also confers resistance to antibiotics. Therefore, the ability of biofilm formation was measured in ARSS and ASSS. The *A*_570_ values of the ASSS group were remarkably reduced with a 2-fold decrease compared to the ARSS group (Figure 3B, *P* < 0.05).

### The mRNA Transcript Levels of Virulence Genes

**Figure 4:**
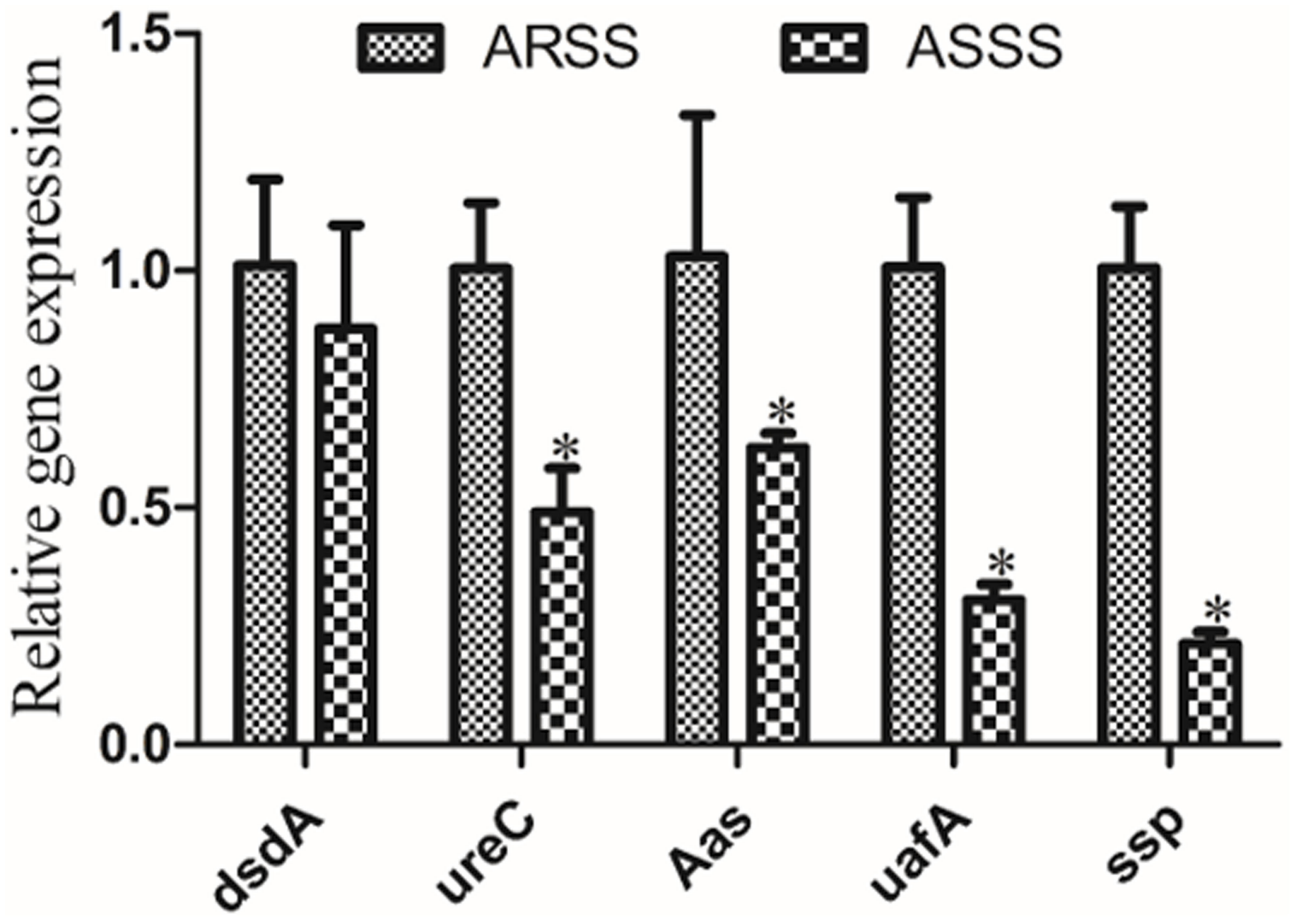
RT-PCR measurement of the differentially virulence-associated genes in the ARSS and ASSS strains. Five genes, i.e., *dsdA, ureC, Aas, uafA*, and *ssp*, were selected and amplified using RT-PCR. The 1*6S rRNA* gene was used as the internal control. Significant differences are marked by asterisks (*p < 0.05).

To investigate the changes of the mRNA transcript levels of virulence genes, RT-PCR was carried out. Compared to the ARSS strain, the transcript levels of *ureC, Aas, uafA*, and *ssp* genes of the ASSS strain significantly degraded (*P* < 0.05). However, the RT-PCR data of *dsdA* gene manifested that no pronounced differences were observed between the ARSS and ASSS groups (Figure 4, *P* > 0.05).

### The Clinical Curative Effect of Azm to ASSS or ARSS-Associated Infection

**Figure 5:**
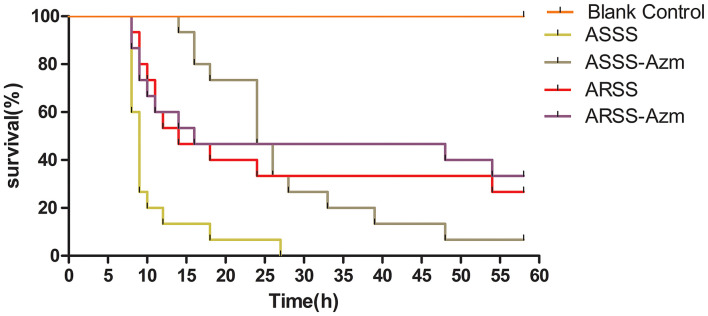
The survival rate of mice infected by the ARSS or ASSS strains. Mice were i.p. injected with the indicated strains, and the mice were monitored for mortality up to 58 h.

The clinical curative effect of Azm is presented in Figure 5. In the ASSS group, infection with the ASSS strain resulted in rapid mouse death, with a 0% survival rate occurring within 25 h. However, in the ASSS-Azm group, the first death occurred at 14 h postinfection, and approximately 6.7% of mice remained alive at 58 h. However, the survival rate of the ARSS-Azm group (33.3%) was similar to that of the ARSS group (26.7%).

### The Changes of Bacterial Burdens

**Figure 6:**
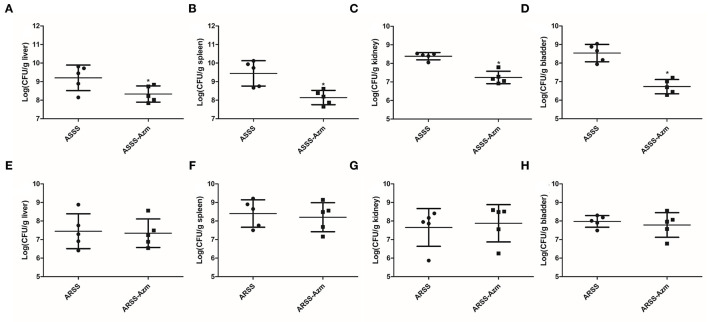
*In vivo* bacterial distribution and burden in organs of mice infected with the ARSS or ASSS strain. At 8 h postinfection, liver **(A,E)**, spleen **(B,F)**, kidney **(C,G)**, and bladder **(D,H)** were collected for measuring the total number of CFU. The number of CFU from each organ was calculated as per gram of tissue. Bars represent the means for five infected mice. Error bars represent standard deviation (SD). Significant differences are marked by asterisks between groups (*p < 0.05).

In order to explore the influences of Azm during ASSS or ARSS infection, the bacterial burdens were calculated in the liver, spleen, kidney, and bladder. Post 8 h infection, compared to the ASSS group, the proliferation level of the ASSS strain in the ASSS-Azm group was significantly reduced (Figures 6A–D). Additionally, the bacterial distribution data showed that no statistically obvious difference was found between the ARSS and ARSS-Azm groups (Figures 6E–H). These results revealed that Azm efficiently inhibited the proliferation of the ASSS strain in the tissues rather than the ARSS strain.

### Pathological Changes of the Kidney

**Figure 7:**
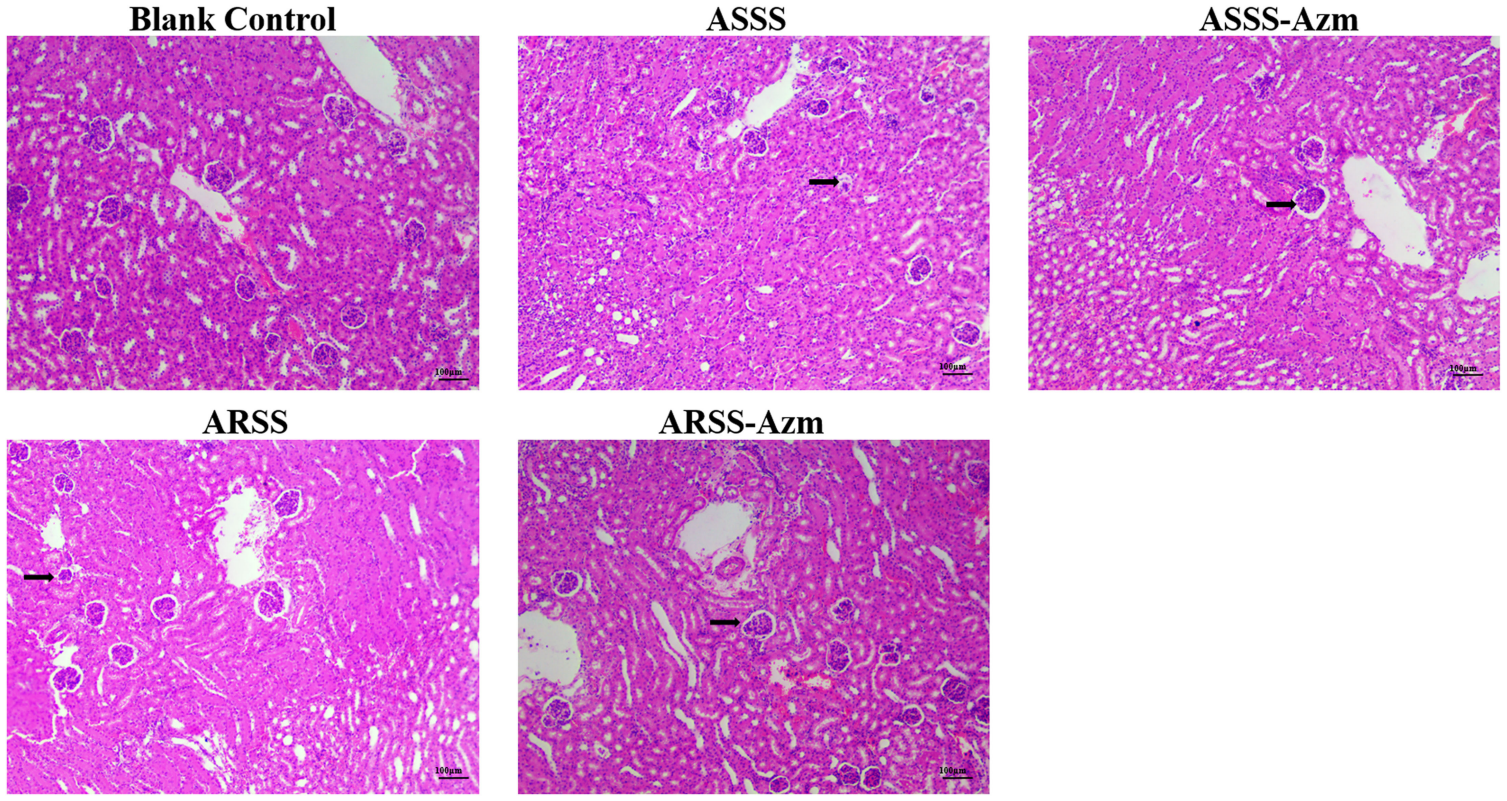
Kidney histological changes of each group at 8 h postinfection (HE stain, ×200). Kidney tissues were randomly isolated and collected from five mice in each group at 8 h postinfection. A portion of each isolated kidney tissue was instantly fixed in 4% paraformaldehyde, followed by hematoxylin and eosin (H&E) stain. Arrows indicate atrophic glomerulus.

The kidney histological changes of each group observed by HE staining are summarized in Figure 7. As shown in the figures, no lesion was observed in the kidney of the BC group. However, the histopathology of the mice in other groups showed that atrophic glomerulus (indicated by arrows) was observed at 8 h postinfection. Furthermore, kidney histological changes of the ASSS-Azm group were significantly alleviated with an obvious decrease in the atrophic glomerulus. In contrast, the histological changes of the ARSS-Azm group were similar to those of the ARSS group.

### Serum Cytokine Levels

**Figure 8:**
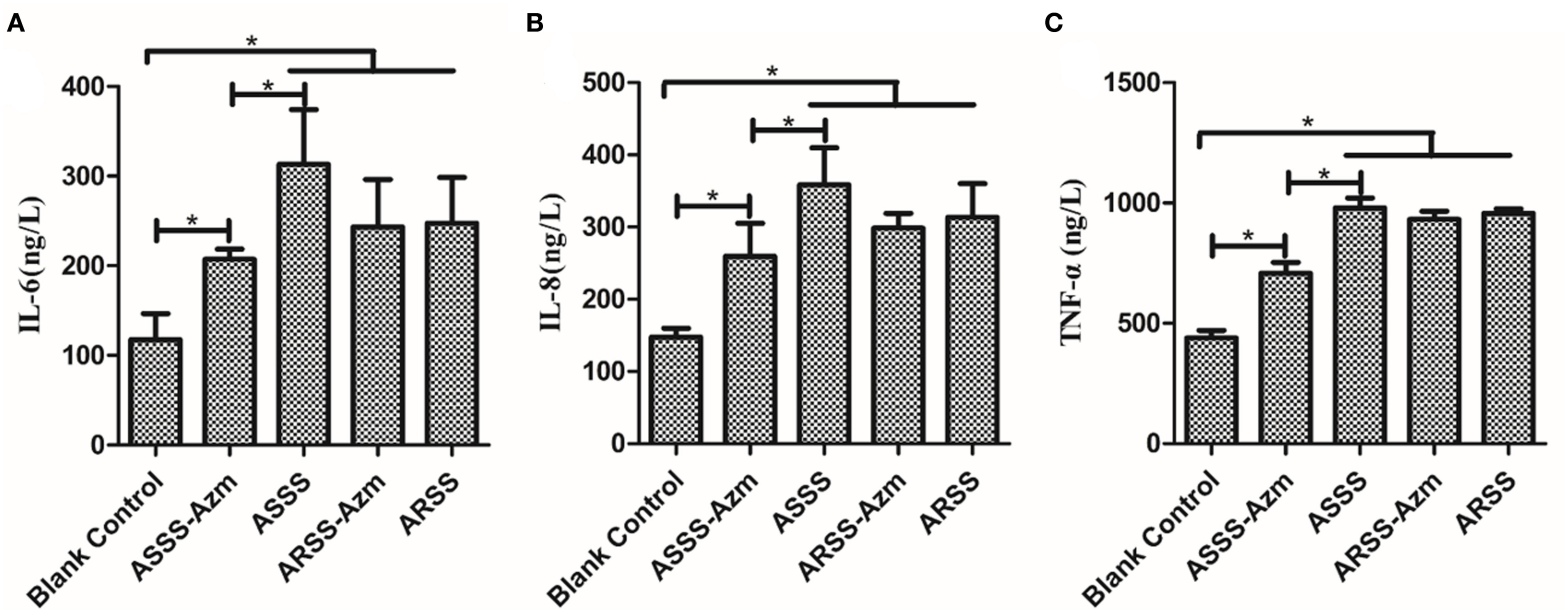
Cytokine levels in the serum of mice i.p. injected with ASSS or ARSS. IL-6 **(A)**, IL-8 **(B)**, and TNF-α **(C)** levels in the serum were measured by ELISA at 8 h postinfection. Values were presented as the mean ± SD. Significant differences between groups are linked by brackets. *p < 0.05.

ELISA was carried out to detect the levels of IL-6, IL-8, and TNF-α in serum at 8 h postinfection. As illustrated in Figure 8, in the BC group, the levels of IL-6, IL-8, and TNF-α were remarkably lower than those in the other groups (*P* < 0.05). Compared with the ASSS group, Azm significantly induced a decrease of IL-6, IL-8, and TNF-α in the ASSS-Azm group (*P* < 0.05). In addition, the results of cytokine levels indicated that there were no pronounced differences between ARSS-Azm and ARSS groups (Figure 8).

## Discussion

Global healthcare was threatened by the rapid emergence and spread of multidrug-resistant bacteria. Coupled with the fact that the development of novel antibiotics is very slow, it is necessary to develop antibiotic adjuvants to reverse antibiotic resistance (9). Bacteria that are previously sensitive to commonly used antibiotics became resistant by stepwise exposure to these compounds, and efflux pump inhibitors could reverse this resistance (20). Our previous investigation demonstrated that Bac could hinder the effect of MsrA efflux pump in ARSS by decreasing the ATP content and the mRNA transcript levels of the *msrA* gene (18). Simultaneously, ARSS recovered the sensitivity to Azm under the exposure of sub-MIC of Bac (Figure 2). The strain that recovered susceptibility was renamed ASSS. The mRNA transcript level of the *msrA* gene in the ASSS strain was lower than the ARSS strain (Figure 3A). What is important is that the rate of resistance reversal was highest in the 31.25-mg/l Bac group (Figure 2A). The reason for the phenomenon is that 62.5–250 mg/l Bac moderately inhibited the growth of ARSS (Figure 1). The MICs of Azm against ARSS and ASSS were identical within 20 passages (Figure 2B). These results reflected that Bac is a potential antibiotic adjuvant of reversing resistance.

The major mechanisms of macrolide resistance to staphylococci involve (i) modification by methylation of 23S ribosomal rRNA, (ii) Msr family efflux pump upregulation, and (iii) macrolide inactivation by phosphotransferases or esterases (21). Also, biofilm is also the main factor influencing the efficiency of antibiotics. In this investigation, the ability of biofilm formation and the transcript levels of *mphC* and *msrA* efflux genes simultaneously decreased in the strain that recovered susceptibility to Azm (ASSS) compared with the ARSS strain (Figure 3). The expression of *mphC* in *S. aureus* was shown to be highly dependent on the presence of a portion of the gene encoding the MsrA efflux pump (22). It has been proposed that the transcriptional level of biofilm matrix components and biofilm formation could be decreased by genetic inactivation and inhibitor of efflux pumps (23). We previously indicated that Bac inhibited biofilm formation by modulating the MsrA efflux pump of ARSS (18). Therefore, we surmised that along with the decrease of transcript levels of the *msrA* gene, the transcript level of the *mphC* gene and biofilm formation ability dwindled under the exposure of sub-MIC of Bac. The mechanisms needed to be further investigated.

At present, the therapeutics of anti-virulence have shown potential in preventing the bacteria from acquiring antibiotic resistance (24). It is reported that Bac suppressed the relative expression of virulence-related genes in *S. aureus* (25) and protected mice from lethal Shiga-like toxin 2 (Stx2) challenge by inducing Stx2 to form inactive oligomers (26). In this study, serial passage of ARSS under sub-MIC of Bac over 20 days failed to produce resistant mutants (Table 2). Simultaneously, compared with the ARSS strain, the mRNA transcript levels of *dsdA, ureC, Aas, uafA*, and *ssp* genes were decreased in the ASSS strain (Figure 4). The activities of ureC and dsdA are essential for successful colonization and pathogenicity in *S. saprophyticus* (15). UafA of *S. saprophyticus* was a cell wall-anchored protein with an LPXTG motif (15). Proteins associated with the surface of *S. saprophyticus* (ssp) (1) and a fibronectin-binding autolysis Aas (16) were non-covalently surface-associated proteins. Therefore, we inferred that the colonization ability and pathogenicity of ASSS were lower than those of ARSS. When mice were infected with ARSS and ASSS in OD_600nm_ of 2.71, the mortalities were 83.3 and 54.0%, respectively (data not shown). Then we extended our study on the protective effects of Azm on the treatment of ASSS or ARSS infections in mice. In the verification experiment, Azm delayed the start time of death, alleviated the injury of the kidney, and decreased the bacterial burden in tissues and cytokine levels in mice infected with ASSS. In contrast, Azm could not treat the infection caused by the ARSS strain (Figures 5–8). These results suggested that Azm exhibited a better therapeutic efficacy on ASSS infection than ARSS infection.

## Conclusion

In conclusion, the sub-MIC of Bac reversed the resistance of ARSS to Azm. The strain that recovered sensitivity to Azm after being exposed to sub-MIC of Bac was named ASSS. The ability of biofilm formation and the mRNA transcript levels of *msrA, mphC*, and virulence genes were decreased in the ASSS strain. Azm successfully treated the infection caused by ASSS rather than ARSS. These results indicated that Bac could be expected to be developed into a new adjuvant of resistance reversal drug.

## Data Availability Statement

The original contributions presented in the study are included in the article/supplementary material, further inquiries can be directed to the corresponding author/s.

## Ethics Statement

The animal study was reviewed and approved by Institutional Animal Care and Use Committee (IACUC) set by Nanjing Agricultural University (approval number: PTA030). Written informed consent was obtained from the owners for the participation of their animals in this study.

## Author Contributions

JW, JM, YW, and JL designed the experiment. JW, JZ, TQ, JD, and WW conducted the research. JW, ZL, and KL analyzed the data and wrote the manuscript. YW and DW revised the manuscript. The final manuscript has been read and approved by all authors.

## Funding

This research was financially supported by the National Natural Science Foundation of China (NSFC, Grant No.31872514).

## Conflict of Interest

The authors declare that the research was conducted in the absence of any commercial or financial relationships that could be construed as a potential conflict of interest.

## Publisher's Note

All claims expressed in this article are solely those of the authors and do not necessarily represent those of their affiliated organizations, or those of the publisher, the editors and the reviewers. Any product that may be evaluated in this article, or claim that may be made by its manufacturer, is not guaranteed or endorsed by the publisher.

## Abbreviations

ARSS: Azm-resistant *S. saprophyticus*
ASSS: Azm-sensitive *S. saprophyticus*
Azm: azithromycin
Bac: baicalin.
